# Surgical management of pulmonary hydatid cysts in children in KwaZulu-Natal Province, South Africa

**DOI:** 10.7196/AJTCCM.2020.v26i3.108

**Published:** 2020-10-13

**Authors:** K Ngcobo, R Madansein, M Ndlovu, R Masekela

**Affiliations:** 1 Department of Cardiothoracic Surgery, Inkosi Albert Luthuli Central Hospital, College of Health Sciences, Nelson R Mandela School of Medicine, University of KwaZulu-Natal, Durban, South Africa; 2 Department of Maternal and Child Health, Inkosi Albert Luthuli Central Hospital, College of Health Sciences, Nelson R Mandela School of Medicine, University of KwaZulu-Natal, Durban, South Africa

**Keywords:** Intact/simple cyst, Ruptured/complicated cyst, Enucleation, Capitonnage, Prolonged air leak

## Abstract

**Background:**

Pulmonary hydatid cyst disease is still a major problem in countries like South Africa (SA), where livestock farming is common. Hydatidosis has a variable clinical course depending on the size, location and complications of the cyst. For pulmonary cysts of any size, surgery remains the gold standard for treatment, with lung conservation surgery being the ideal.

**Objectives:**

To describe the epidemiology, clinical presentation, surgical management, and surgical outcomes of paediatric pulmonary hydatid disease in children referred to the Department of Cardiothoracic Surgery at Inkosi Albert Luthuli Central Hospital (IALCH) in KwaZulu-Natal Province, SA.

**Methods:**

We retrospectively reviewed and analysed the medical records of 38 children between the ages of 0 and 18 years with pulmonary hydatid cysts, who were referred to the Department of Cardiothoracic Surgery at IALCH and underwent surgical management between 1 January 2008 and 31 December 2018. The medical records were evaluated for patients’ demographics, clinical evaluation, surgical management strategies and surgical outcomes.

**Results:**

Of the 38 patients, 60.5% were male, with a mean age of 6.5 years. More than two-thirds of the patients (68.4%) were from the Eastern Cape and 31.6% were from KwaZulu-Natal. The majority of the cysts (84.2%; n=32) were classified as large (5 - 9 cm in diameter) and giant (≥10 cm in diameter). Forty-eight surgical procedures were performed and lung preservation surgery by enucleation and capitonnage was preferred. Some patients developed postoperative complications such as prolonged air leaks from bronchopleural fistula (18.8%; n=9) and 88.9% (n=8) of these patients healed with chest tube drainage and physiotherapy. Lung resection was only required in 4% (n=2) of the patients. The mean (standard deviation) number of days spent in hospital was 7 (4) days. All children survived with no recurrences.

**Conclusion:**

Conservative surgical procedures such as enucleation of the cysts of any size are possible, safe, reliable and reproducible.

## Background

Hydatid disease is a common health problem, especially in regions where livestock (goats, sheep and cattle) and humans live in close proximity, e.g. South America, Australia, India, the Middle East, sub-Saharan Africa and Mediterranean countries.^[1–3]^ South Africa (SA) has a large rural community where livestock subsistence farming is common. Hydatid disease is caused by a parasite, *Echinococcus granulosus*, that accidentally infects humans. Dogs and other canines are the definitive hosts while livestock, including cattle, sheep, goats and pigs, are the intermediate hosts.^[2,4]^ Four species of *Echinococcus* have been identified (recent literature states six species are recognised); however, *E. granulosus* and *E. multilocularis* are the most important species that are infective to humans and cause cystic and alveolar echinococcosis, respectively.^[1,5]^

The World Health Organization estimates that >1 million people become infected with *Echinococcus* spp. annually worldwide. ^[1,4]^ The epidemiology of hydatid disease is not known in SA. A retrospective data analysis of the National Health Laboratory Service information system on echinococcosis serology, microscopy and histopathology results in eight provinces (excluding KwaZulu-Natal (KZN)) revealed an overall positivity rate in submitted diagnostic samples of 17.0% (n=1 056/6 211).^[1]^ The Eastern Cape (EC) and Northern Cape provinces had infection rates of 30.4% and 18%, respectively.^[1]^

Hydatidosis has a variable clinical course. It may be asymptomatic for many years and only become evident when a cystic lesion is noted incidentally during imaging for other reasons or it may be symptomatic depending on the size, location and complications of the cyst.^[2,4,6]^ Hydatid cysts can affect any organ in the human body but the liver and lungs are the most frequently involved organs. In children, the lungs are the most commonly affected organ.^[1,3,7]^ Symptoms of pulmonary hydatid disease include cough, chest pains, fever, dyspnoea and rarely expectoration of salty material.^[3,5]^ However, the rupture of the cyst into either the airways or pleural space may result in haemoptysis or pleural effusion, which usually complicates into an empyema. Chest radiography and computed tomography (CT) scan of the chest and upper abdomen are usually the imaging modalities used to achieve the diagnosis of pulmonary hydatidosis.^[2,8,9]^

Many serological laboratory tests are available for the diagnosis of echinococcosis; however, a negative test does not necessarily exclude pulmonary hydatidosis. Serological tests usually yield positive results if a patient has multiple cysts or a ruptured pulmonary cyst, but they are not very sensitive for detection of isolated pulmonary hydatid cysts (PHCs).^[5]^ Serology may also be used for patient follow-up as an indicator of relapse, recurrence and if there is doubt on preoperative imaging.^[4]^

Lung-sparing surgery is the gold standard for the treatment of PHCs.^[2-8,10]^ In SA and the broader African continent, there are limited data on pulmonary hydatid disease in both adults and children.

## Methods

This was a retrospective observational study that was done on children with PHCs from a single centre in KZN Province, SA. Children were included if they were 0 - 18 years of age and were referred to the Department of Cardiothoracic Surgery at the Inkosi Albert Luthuli Central Hospital (IALCH), a quaternary hospital, between 1 January 2008 and 31 December 2018. IALCH accepts referrals from KZN and EC provinces for surgical management. There are 2.47 million (60%) and 1.66 million (63%) children living in the rural areas of KZN and EC, respectively.^[1]^

The age, gender, symptoms, radiographical findings of the cysts (location, size and nature of cysts (ruptured or intact)), surgical procedures, morbidity (complications and length of hospital stay), mortality and recurrence of pulmonary cysts were obtained from patients’ files. The routine surgical procedure in the current study was enucleation with closure of bronchial openings and capitonnage of the residual cyst space, which is a lung-conserving approach. All patients received albendazole postoperatively, which was administered at 20 mg/kg/day for 2 weeks if the cyst was intact and for 4 - 6 weeks for ruptured or multiple cysts. All patients were followed-up at 6 weeks, 3 months, 6 months and 1 year and symptoms and chest radiographs were evaluated.

### Operative techniques

All procedures were performed under general anaesthesia, with a single-lumen endotracheal tube and the affected lung was isolated using a bronchial blocker. All patients had a posterolateral thoracotomy. Patients with bilateral PHCs had a second posterolateral thoracotomy on the contralateral side 6 - 8 weeks after the initial procedure. In the present study, removal of the unruptured cyst was done first if there were bilateral cysts where one had ruptured, and if both sides had intact cysts, the larger cyst was removed first. Our preferred surgical procedure was enucleation with capitonnage if the cyst was uninfected. After a standard posterolateral thoracotomy, the hydatid cyst was identified and the surgical wound and adjacent tissue were covered with packed gauze soaked in 5% hypertonic saline solution so that the only area exposed is the lung containing the hydatid cyst. Hypertonic saline has scolicidal properties that help to limit contamination and spillage should the PHC rupture during removal, thus prevent the development of a secondary hydatid cyst. Removal of the intact cyst using enucleation (Ugon method) was done by carefully making an incision in the pericyst and then utilising positive-pressure ventilation to deliver the intact cyst (Figures 2 and 3). The residual cavity was washed with saline and all bronchial openings that were identified were closed with prolene sutures. The same was done with any bleeding vessels. Capitonnage was performed to obliterate the cyst space. In patients with ruptured and/or infected complicated cysts, after making an incision in the pericyst, the germinative membrane was removed and the cystic cavity was carefully cleaned by suction and washed with 5% hypertonic saline; thereafter, bronchial openings were identified and closed. Capitonnage was not done for infected cysts. Albendazole was administered at a dose of 20 mg/kg/day postoperatively for multiple cysts, ruptured cysts and if there were abdominal cysts.

### Statistical analysis

Data were collected and captured in Microsoft Excel and analysed using SPSS version 25 for Windows (IBM Corp., USA). χ2 was conducted using custom tables for age, gender, indirect haemagglutination assay (IHA), postoperative complications and length of hospital stay and each variable was compared with the type of cyst (intact or ruptured). Both age and gender were also tested for whether they influenced the size of the cyst as well as surgical outcomes. Surgical outcomes (postoperative complications and hospital stay) were compared with the surgical procedure that was done. In all cases, the results were considered statistically significant if p<0.05. Ethical approval was granted by the Biomedical Research Ethics Committee of the University of KwaZulu-Natal (ref. no. BREC 1271/2020).

## Results

In total, 38 patients were included in the study. The children were predominantly male (60.5%; n=23) and the mean age (range) was 6.5 (1 - 12) years. All participants were of black African ethnicity and the majority (n=36/38) of them had been exposed to dogs, sheep and cattle. The source of exposure for the remaining 2 patients was not recorded and was unknown. Most children (92%; n=35) presented with a cough, fever or both; 2 were asymptomatic (5.3%) and 2.6% (n=1) presented with abdominal pain. (Table. 1) depicts the preoperative characteristics of the patients with PHC. Age and gender had no significant correlations with intact or ruptured cysts (Table. 2). Both age (p=0.310) and gender (p=0.423) did not influence PHC size (Table. 2). The distribution of cysts is depicted in (Table. 3).

**Fig. 1:**
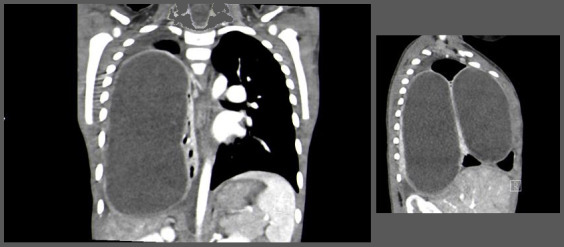
Computed tomography scan showing two giant pulmonary cysts in the right lung of 13 × 8 × 7 cm (posterior cyst) and 11 × 7 × 6 cm (anterior cyst).

The rupture of PHCs did not affect the serological outcome; however, there was a significant association between multiple cysts and positive serology (p=0.035). All cases were proven to have hydatid disease by histopathological analysis of the pulmonary cyst specimen sent postoperatively. The sensitivity of IHA was 64.3% in the current study. All the participants were diagnosed using the chest X-ray and computed tomography (CT) scan of the chest and the upper abdomen. The number of patients that had cysts that were ≥10 cm in dimension (giant cysts) (Fig. 1) was 21.1% (n=8), but the majority of the cases (63.2%; n=24) had cysts that were between 5 and 9 cm and the rest of the cysts were small (<5 cm). Nearly half of the patients with ruptured cysts (47.6%; n=10/21) presented with the classic radiographical signs.

A total of 48 surgical procedures were performed on study participants and 10 patients had bilateral thoracotomies that were 6 - 8 weeks apart for bilateral pulmonary hydatid cysts. Enucleation with capitonnage was done in the majority of cases (83.3%; n=40). Enucleation only was done in 10.4% (n=5) patients and capitonnage was not done when the cyst was infected. One patient underwent pericystectomy, which involved the removal of all three layers of the hydatid cyst. One patient had a left-upper lobectomy because during surgery the left upper lobe was too small and did not re-aerate.

Postoperative complications were observed in 33.3% (n=16) of the cases (Table. 4). Prolonged lung air leaks (>5 days) were observed in 18.8% (n=9) of the patients and one of these had a re-thoracotomy 10 days post operation for bronchopleural fistula repair, which was unsuccessful due to failure of expansion of the lung and resulted in a need for a left pneumonectomy. Lung resection was done in only 4.2% (n=2) of the participants. All other air leaks (16.7%; n=8) were managed successfully with chest physiotherapy and chest tube drainage. Atelectasis developed in 6.2% (n=3) of the patients – 2 of these resolved after a few days of aggressive chest physiotherapy and the last one resolved after bronchoscopy, mucus plug removal and secondary chest physiotherapy. One patient had a ruptured splenic cyst which was intact at preoperative assessment and a laparoscopic splenectomy was done 2 days post enucleation of the lung cyst.

Two (4.2%) patients had pneumothorax post chest drain removal, both of which resolved with conservative management. One patient with an infected cyst developed an empyema thoracis that was managed with antibiotics and chest tube drainage. The child recovered and was discharged after 14 days post operation. There was no significant association between the type of procedure and postoperative complications (p=0.054).

Of the complications, 81.3% (n=13/16) occurred in participants with ruptured cysts (p=0.009). The mean (SD) hospital stay was 7 (4) days. More than two-thirds of the patients (70%) had hospital stays of 7 days or less, while 29.9% of the patients stayed for >7 days. There was no significant association between type of surgery and length of hospital stay (p=0.207).

Patients who developed complications after surgical management of PHC were more likely to have a prolonged hospital stay (p<0.05). The type of procedure done did not impact the duration of hospital stay.

## Discussion

The current study showed a male predominance and the majority of patients came from the EC Province. Enucleation and capitonnage resulted in 75% of the participants having a good outcome with no postoperative complications and 70% were discharged home within 7 days of hospital admission. There was no mortality reported and no recurrence after a year of follow-up. Only 2 participants required lung resection.

The laboratory assay, particularly the *Echinococcus* immunoglobulin (IgG) assay used in this study, still remains a poor screening tool for PHC. Serology is likely to be positive when multiple cysts are present, not for an isolated cyst.^[5]^ Exposure to animals such as cats, dogs, sheep and cattle is a well-known risk factor for hydatid disease.^[8]^

Most of the cases (94.7%) in this study were from the rural areas of KZN and EC provinces and had a history of exposure to these animals. Only one case was from an urban area of KZN.

In children, hydatid cysts occur mainly in the lungs and less often in the liver; however, in adults, the opposite is true.^[3,8,9]^ Only 10 patients had liver cysts in the current study. Patients with intact cysts are usually asymptomatic; however, some do develop symptoms such as cough, dyspnoea, fever and chest pain.^[4]^

A chest radiograph is the first radiological analysis that is performed for all patients with pulmonary hydatid disease and its diagnostic value for intact cysts is very high.^[8]^ A contrast-enhanced CT scan of the chest and upper abdomen should be performed to confirm the diagnosis, identify liver and splenic cysts and help with the planning of the necessary surgical procedure. An intact hydatid cyst appears as a well-defined homogenous opacity on a chest radiograph, of which the differential diagnoses include fluid-filled cysts, benign and malignant tumours, metastases and inflammatory masses.^[11]^ The classic radiographical signs of a complicated cyst include a crescent sign, waterlily sign, serpent sign and many others which are not always present.^[11–13]^ All patients in this study had a contrast-enhanced CT scan of the chest and upper abdomen. Children have more elastic lung tissues, tend to have larger cysts and delayed presentation.^[4]^ Furthermore, they may present with so-called giant cysts (>10 cm in size).^[4]^

**Fig. 2:**
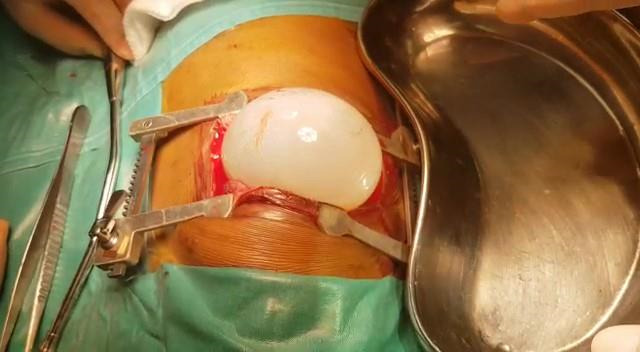
Enucleation of intact cyst via right posterolateral thoracotomy.

**Fig. 3:**
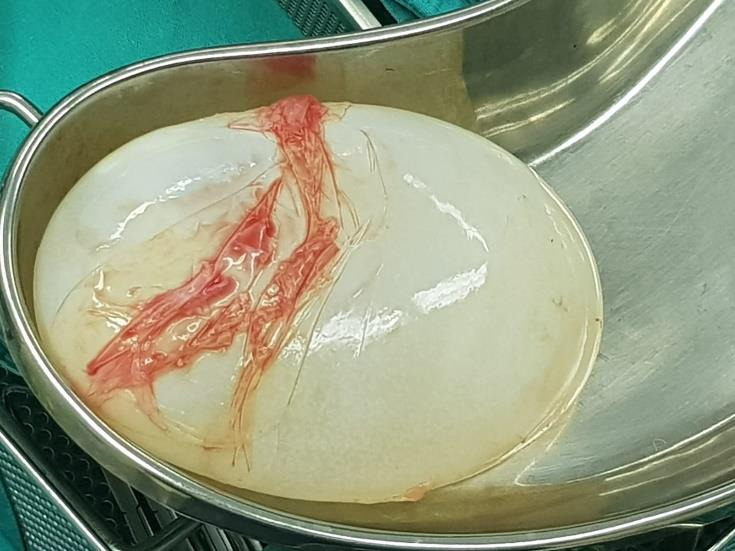
Giant cyst after enucleation.

In a study of 1 055 patients in Turkey,^[11]^ most cysts were found in the lower lobes compared with upper lobes and in the right lung compared with the left lung. Studies have reported that ~30% of patients with PHCs can have multiple cysts while about 20% have bilateral cysts.^[4]^ This is in agreement with what we observed in our study, where the number of patients with multiple and bilateral cysts was 33% and 26%, respectively. The majority of the cysts (71.1%) were in the right side of the lung and lower lobes were more commonly affected, in line with the literature.^[3,4,8-10]^

The gold standard for managing PHCs of any size is lung-conservation surgery. This ensures that the maximum amount of viable lung is maintained while ensuring complete removal of all viable parasite material.^[2-8,10]^ Different surgical procedures have been described in the literature, such as enucleation (Ugon method) with or without capitonnage,^[4]^ pericystectomy (Perez‑Fontana method),^[5]^ cystotomy with capitonnage (Barrett’s method),^[2]^ cystostomy with the closure of the bronchial openings alone,^[11]^ open aspiration by the Figuera technique,^[10]^ and lung resection (i.e. wedge resection, segmentectomy and lobectomy).^[3,5]^ We used enucleation and capitonnage whenever possible. There is a lack of consensus in the literature about the use of capitonnage, with some studies showing no benefit,^[4,15]^ while others have shown excellent outcomes.^[3,8,10,14]^ There is consensus among surgeons about the closure of bronchial openings and this is seen as an important step in any lung-conserving approach during PHC surgery.

We opted for staged thoracotomies in the presence of bilateral pulmonary hydatid disease. Median sternotomy or staged thoracotomies have been reported to yield good results in the literature.^[5,10,11]^ However, patients have to be carefully selected for median sternotomy.

The literature has demonstrated that in patients with both intact or ruptured bilateral hydatid cysts, it is better to first operate on the side with a large cyst and then later follow on the side with a smaller cyst. ^[15,16]^ In patients with bilateral cysts where one cyst has ruptured and the patient tolerates the ill-effects of the rupture, the intact cyst should be removed first to minimise the risk of its rupturing. In the current study, 7 out of 10 patients with bilateral PHCs had complications during the first or second operation.

Previous studies have shown that anti-helminthic agents impair the cyst membrane and may lead to cyst rupture if these agents were administered preoperatively.^[8]^ In fact, some studies have reported the incidence of cyst rupture due to albendazole to be ~70%.^[8]^ This is why surgeons at the Department of Cardiothoracic Surgery at IALCH do not recommend administration of anti-helminthic treatment for patients with intact cysts.

Factors such as the size and number of the cysts influences the patient’s response to therapy, with favourable responses observed in patients with smaller cysts.^[17]^ We found that some patients in this study were started on medical therapy before contact with cardiothoracic surgeons by their consulting physicians. Surgeons at the Department of Cardiothoracic Surgery at IALCH prefer surgical management first, followed by medical therapy after the operation. There is a risk for hydatid cyst to recur; thus, some surgeons advocate for postoperative medical therapy to reduce the risk of recurrence.^[4,5,8,18]^ There were no recurrences and mortality in this study; however, the follow-up period was short. Previous studies have found that recurrence rates varied between 4.6% and 22% after surgical management.^[4]^ In contrast, a study by Bagheri *et al*. ^[15]^ found a recurrence rate of 2.5% in 1 024 patients who were followed-up for a period of 2 - 24 years.^[15]^ Many studies have demonstrated that the mortality rate from PHC surgery is negligible.^[4,5,11,15,18]^

The strength of this study is that it is the first study to our knowledge that has analysed the surgical outcomes in children with PHCs from KZN and EC provinces. The limitations of the study are that this is a retrospective study with a short follow-up period and a small sample size. Therefore, a larger study is required to validate our findings.

## Conclusion

Conservative surgical procedures such as enucleation of cysts of any size are possible, safe, reliable and reproducible. These result in manageable complications and negligible mortality. Healthcare education and prevention programmes are needed in the rural communities to prevent pulmonary hydatid cysts.

## Figures and Tables

**Table 1:** Preoperative diagnosis of hydatid cysts

Variable	Single cyst, *n*	Multiple cysts, *n*	Total, *n*	*p*-value
IHA				0.035
Positive	5	13	18	
Negative	8	2	10	
Not recorded	5	5	10	
Total	18	20	38	

IHA = indirect haemagglutination assay

**Table 2:** Characteristics of patients with cysts

Variable	Intact cysts, *n* (%)	Ruptured cysts, *n* (%)	Total, *n* (%)	*p*-value
Age (years)				0.202
<5	8 (47.1)	5 (23.8)	13 (34.2)	
5 - 10	8 (47.1)	11 (54.4)	19 (50.0)	
>10	1 (5.8)	5 (23.8)	6 (15.8)	
Gender				0.509
Male	9 (52.9)	14 (66.7)	23 (60.5)	
Female	8 (47.1)	7 (33.3)	15 (39.5)	
IHA				0.473
Positive	7 (41.2)	11 (52.4)	18 (47.4)	
Negative	5 (29.4)	5 (23.8)	10 (26.3)	
Unknown	5 (29.4)	5 (23.8)	10 (26.3)	

IHA = indirect haemagglutination assay

**Table 3:** Anatomical location of hydatid cysts and frequency (*N*=38)

Location of cyst	*n *(%)	
Right upper lobe	9 (23.7)
Right middle lobe	4 (10.5)
Right lower lobe	14 (36.9)
Left upper lobe	7 (18.4)
Left lower lobe	4 (10.5)
Bilateral (right and left)	10 (26.3)
Liver involvement	8 (21.1)

**Table 4:** Postoperative outcomes of pulmonary hydatid cysts

Variable	E & C, *n* (%)	Enucleation, *n* (%)	Other*, *n* (%)	Total, *n* (%)	*p*-value
Complications					0.054
Prolonged air leaks	5 (12.5)	2 (40.0)	2 (66.7)	9 (18.8)	
Atelectasis	2 (5.0)	0	1 (33.0)	3 (6.3)	
Pneumothorax	2 (5.0)	0	0	2 (4.2)	
Empyema	0	1 (20.0)	0	1 (2.0)	
Splenic cyst rupture	1 (2.5)	0	0	1 (2.0)	
No complications	30 (75.0)	2 (40.0)	0	32 (66.7)	
Length of hospital stay (days)					0.207
≤5	20 (50.0)	1 (20.0)	0	21 (43.8)	
6 - 7	12 (30.0)	1 (20.0)	0	13 (27.1)	
>7	8 (20.0)	3 (60.0)	3 (100)	14 (29.1)	
Mortality	0	0	0	0	NA
Recurrence at 1 year	0	0	0	0	NA

E & C = enucleation and capitonnage

NA = not applicable

^*^Other represents pericystectomy, lobectomy and pneumonectomy
